# Clinical and radiological outcomes of lumbar endoscopic decompression for treating lumbar spinal stenosis and degenerative lumbar scoliosis: a retrospective study at mean 4.4 years follow-up

**DOI:** 10.3389/fsurg.2024.1525843

**Published:** 2025-01-15

**Authors:** Ning Fan, Aobo Wang, Shuo Yuan, Peng Du, Tianyi Wang, Lei Zang

**Affiliations:** Department of Orthopedics, Beijing Chaoyang Hospital, Capital Medical University, Beijing, China

**Keywords:** degenerative lumbar scoliosis, lumbar spinal stenosis, endoscopy, outcomes, complications

## Abstract

**Purpose:**

To assess the clinical and radiological outcomes of lumbar endoscopic decompression for the treatment of lumbar spinal stenosis (LSS) with concurrent degenerative lumbar scoliosis (DLS).

**Methods:**

This study retrospectively reviewed 97 patients with LSS and DLS who underwent lumbar endoscopic decompression between 2016 and 2021. The average follow-up duration was 52.9 months. Another 97 LSS patients without DLS were selected as the control group. The pre- and postoperative visual analog score (VAS) and the Oswestry disability index (ODI) were recorded and analyzed to compare clinical outcomes. Radiological findings, such as coronal balance and intervertebral disc height, have also been reported.

**Results:**

Both groups' mean VAS scores for back pain, leg pain, and ODI were significantly improved two weeks after surgery and at the final follow-up (*p* < 0.001). There was no significant difference in the prevalence of surgical complications or patient satisfaction rates. However, patients in the DLS group reported more severe back pain at the final follow-up than those in the LSS group (*p* = 0.039). Radiological follow-up revealed no significant deterioration in coronal imbalance or loss of disc height in either group.

**Conclusion:**

Lumbar endoscopic decompression can be a safe and effective surgical technique for treating LSS with DLS, particularly in elderly patients with poor general conditions.

## 1 Introduction

With the advancement of social age, degenerative lumbar scoliosis (DLS) has become a common adult spinal deformity (1). DLS is defined as lumbar spine curvature, also known as Cobb's angle, greater than 10° in a skeletally mature individual (2). The prevalence of DLS ranges from 6%–68% (3). In terms of etiology and pathology, DLS is closely linked to intervertebral disc degeneration, facet joint arthritis, and paraspinal muscle degeneration, all of which contribute to lumbar instability and intervertebral space collapse (4). Consequently, this frequently causes the onset or worsening of lumbar spinal stenosis (LSS), which is characterized by symptoms such as radiculopathy and neurogenic claudication (5).

The widely accepted gold standard for treating LSS with DLS is lumbar decompression and fusion. This procedure allows for complete decompression, spinal stabilization, and deformity correction if necessary (6, 7). However, traditional surgeries require extended incisions, disruption of the paraspinal structures, and instrumentation, all of which increase the risk of complications related to internal fixation, iatrogenic instability, and adjacent segment degeneration. Therefore, the necessity of fixed fusion has been debated in recent years. For the treatment of other types of lumbar instability such as spondylolisthesis, evidence suggests that non-fusion procedures, particularly minimally invasive decompression, are effective alternatives to traditional surgery with comparable efficacy and safety profiles (8–10). This technique avoids disrupting the lumbar stabilizing structures while minimizing surgical trauma and complications. Therefore, it is particularly suitable for elderly patients with predominant radicular symptoms. However, the applicability of this strategy for DLS patients remains uncertain due to limited supporting evidence.

In recent years, with the advancement of minimally invasive techniques, endoscopic decompression surgeries, such as transforaminal decompression, have been recognized for their efficacy in treating various types of LSS (11, 12). However, there remains debate regarding whether this technique is suitable for patients with DLS. Thus, a retrospective cohort study was conducted to examine the clinical and radiological outcomes of lumbar endoscopy in the treatment of LSS with DLS. For comparison, the efficacy of this technique in treating patients with LSS alone has also been evaluated.

## 2 Material and methods

### 2.1 Patient selection

This study retrospectively evaluated the clinical data of 97 consecutive patients with LSS and DLS who underwent unilateral, single-level lumbar endoscopic decompression at our institution between January 2016 and November 2021 (DLS group). The inclusion criteria were as follows: (1) age >40 years; (2) a Cobb's angle >10°; (3) the primary complaint is unilateral radiating pain or numbness in the lower extremity, and combined with imaging findings and physical examination, the diagnosis was lateral recess or foraminal stenosis; and (3) failure of conservative treatment for at least three months. The exclusion criteria were as follows: (1) patients with low back pain as the primary symptom; (2) patients who underwent dual or multilevel lumbar decompression; (3) a distance of more than one level between the responsible level and the scoliosis end vertebra; (4) significant sagittal or coronal instability, or other clear indications for lumbar internal fixation; (5) a follow-up duration of less than 24 months; (6) insufficient clinical or imaging data; and (7) lumbar spine comorbidities, such as fractures, infections, and tumors. To further validate the surgical efficacy of lumbar endoscopic decompression in treating LSS with DLS, a control group (LSS group) of 97 patients with LSS without DLS was established. Due to the extended study duration, the control group was matched based on surgery dates to minimize the potential impact of variations in medical conditions, surgical techniques, and follow-up periods on the treatment outcomes. This study was conducted in accordance with the Declaration of Helsinki and approved by our institutional ethics committee.

### 2.2 Surgical technique

In this study, the percutaneous transforaminal approach was used. The surgeries were performed by a senior surgeon with an experience of more than 100 lumbar endoscopic procedures. In the patients with multilevel radiographic stenoses, diagnositic local blocking was performed to identify the responsible level.

**Figure 1:**
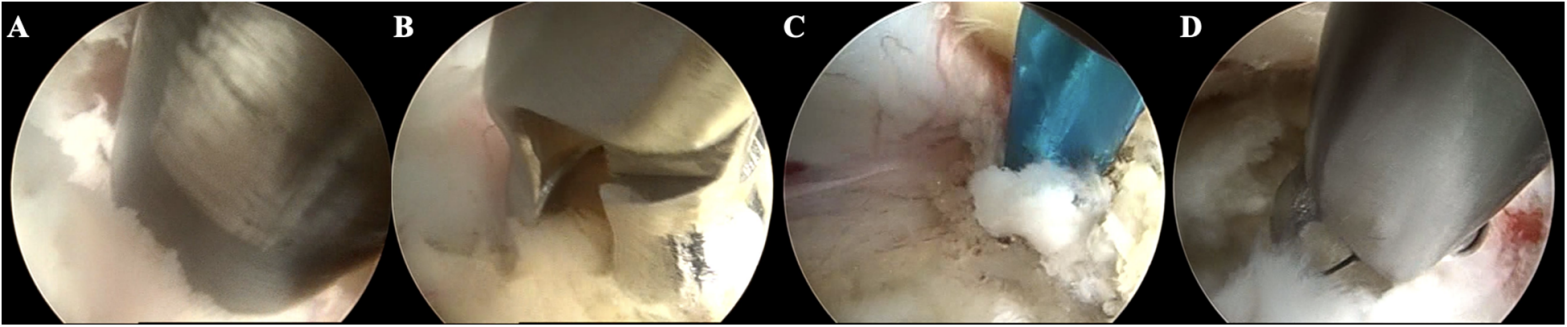
Case illustration of endoscopic exploration in treating LSS with DLS. **(A)** Resecting the articular process using a trephine; **(B)** Removing the hypertrophic ligamentum flavum; **(C)** Separating the adhered nerve root; **(D)** Removing the herniated disc.

The patients were placed in the prone position. Adjusting the angle of the operating table to achieve bilateral symmetry of the pedicles under anteroposterior fluoroscopy, and overlapping of the vertebral posterior margins under lateral fluoroscopy. The entire procedure was performed under local anesthesia. The entry point was set at 10–14 cm lateral to the midline at the index intervertebral level. A puncture needle was inserted into the superior articular process (SAP). An 8 mm working cannula was placed in contact with the surface of the SAP after expending the surgical approach using serial hollow cannulas. A trepan was then used to remove the capsule and ventral side of the SAP to perform foraminoplasty. Decompression was performed using continuous irrigation under direct vision. Based on the endoscopic exploration, a nucleus forceps was used to remove potential factors contributing to nerve root compression, including the thickened ligament flavum, perineural fat, degenerated annulus fibrosus, and herniated nucleus pulposus. A radiofrequency system was also used for ablating the annulus fibrosus compressing the nerve root and providing hemostasis. Then the osteophytes of the posterior margin were carefully removed using a rongeur when neccessary. The traversing nerve root and dural sac were exposed with adequate mobility and good pulse to ensure complete decompression. A case illustration of endoscopic exploration was provided in Figure 1.

### 2.3 Outcome assessment

The clinical symptoms and outcomes of all the patients were evaluated by reviewing their medical records and follow-up data. The visual analog pain score (VAS) and Oswestry disability index (ODI) were scored before surgery, 2 weeks after surgery, and at the final follow-up. The modified MacNab criteria was used to evaluate patient subjective satisfaction, and excellent or good outcomes were considered satisfactory. Additionally, complications from decompression surgeries were also counted.

### 2.4 Radiological assessment

**Figure 2:**
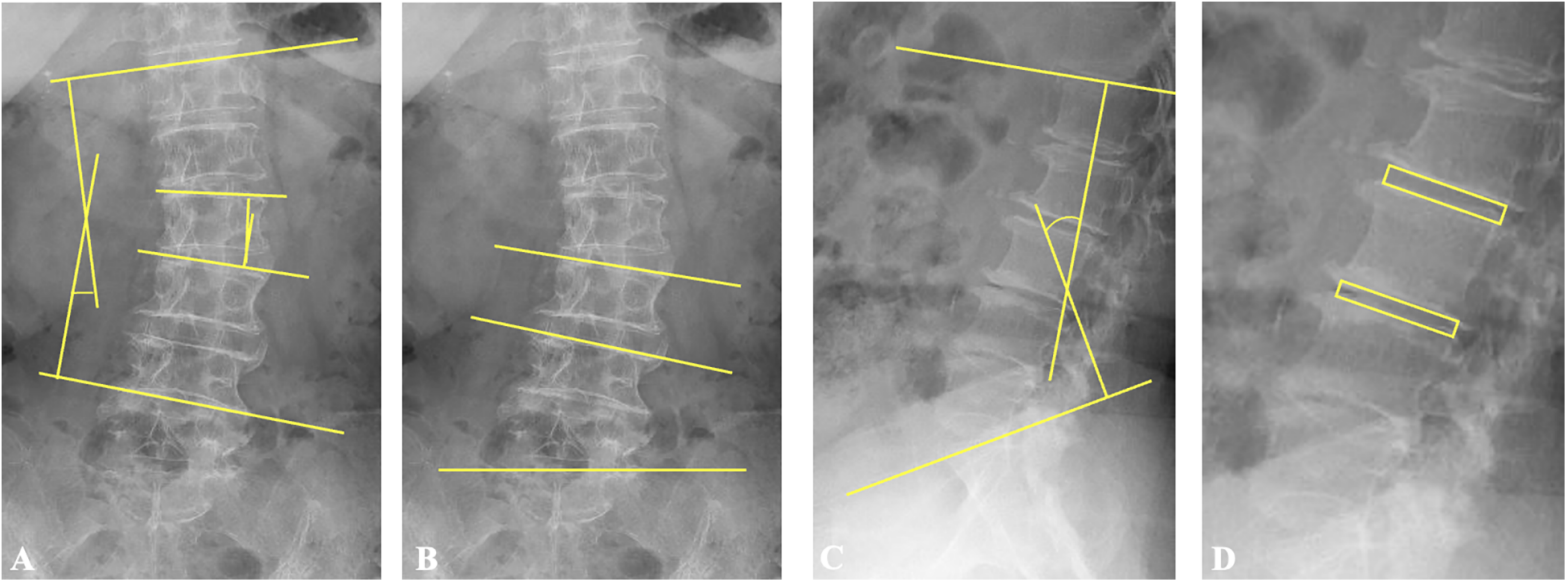
Illustration of the measurements of radiological parameters. **(A)** Cobb's angle is the angle formed by the upper endplate of the upper-end vertebra and the lower endplate of the lower-end vertebra. The segmental coronal angle is the angle formed by the upper endplates of the vertebra below and above the surgical segment. **(B)** L3 and L4 endplate angles denote the angles formed by the upper endplates of L3 and L4, respectively, and the horizontal line. **(C)** Lumbar lordosis is the angle formed by the upper endplate of L1 and the upper endplate of S1 in lateral lumbar spine radiographs. **(D)** Disc height is defined as the average measurement of the anterior and posterior edges of the intervertebral disc at the surgical and adjacent superior levels, respectively.

The radiological outcomes of the patients who underwent lumbar endoscopic decompression were investigated. In this study, 53 patients in the DLS group and 43 patients in the LSS group received postoperative radiological follow-up data for more than one year. Several imaging parameters that are closely related to patients' pain symptoms and lumbar degeneration were chosen, including Cobb's angle, surgical level segmental coronal angle (13), lumbar lordosis, surgical level disc height, proximal adjacent level disc height, L3 endplate angle, and L4 endplate angle (3, 14). Figure 2 illustrates the measurement of these parameters. Pre- and postoperative parameters were compared to confirm the radiological outcomes of lumbar endoscopic decompression.

### 2.5 Statistical analysis

Data were analyzed using the statistical software SPSS (version 23.0 for Windows, IBM). Continuous variables are presented as mean ± standard deviation or median (interquartile range). Independent samples *t*-tests, Pearson's chi-square tests and nonparametric tests for independent samples were used to assess the parameters between the groups. Paired samples *t*-tests and nonparametric tests for related samples were used to compare pre- and postoperative data. Statistical significance was set at *p* < 0.05.

## 3 Results

The medical and follow-up records of 97 patients with LSS and DLS and 97 patients with LSS only were retrospectively reviewed. Table 1 shows the clinical baseline characteristics of all patients. The two groups did not differ significantly in terms of age (70.2 vs. 68.1 years, *p* = 0.148), gender (*p* = 0.061), or follow-up duration (52.9 vs. 53.7 months, *p* = 0.769). However, the DLS group had a significantly higher prevalence of cardiovascular disease than the LSS group (76.3% vs. 53.6%, *p* = 0.001). Furthermore, the responsible level location differed between the two groups (*p* = 0.022). Most patients in the DLS group had nerve root compression at the L3/4 and L4/5 levels, whereas the LSS group's predominant locations were at the L4/5 and L5/S1 levels. Table 2 provides additional information regarding the patients in the DLS group.

**Table 1:** Clinical baseline characteristics of all patients enrolled.

	DLS group	LSS group	*p*
Number of patients	97	97	
Age (years)	70.2 ± 9.4	68.1 ± 10.3	0.148
Gender (male/female)	35/62	49/48	0.061
Comorbidities (*n*)			
Cardiovascular disease	74	52	0.001*
Cerebrovascular disease	23	15	0.148
Endocrine disease	42	33	0.226
Chronic respiratory disease	21	18	0.591
Surgical level			0.022*
L2/3	1	2	
L3/4	13	5	
L4/5	74	69	
L5/S1	9	21	
Follow-up duration (months)	52.9 ± 20.2	53.7 ± 20.8	0.769

* Statistically significant at *p* < 0.05.

**Table 2:** Radiological baseline characteristics of patients in the DLS group.

Cobb's angle (°)	18.1 ± 6.7
Upper end vertebra (*n*)	
T11–12	19
L1–3	78
Lower end vertebra (*n*)	
L4	51
L5	46
Apex vertebra (*n*)	
L1–2	32
L3–4	65
Location of nerve root compression	
Concave side	57
Convex side	42

Table 3 shows the patients' preoperative symptoms and postoperative outcomes. There was no significant difference in the preoperative VAS or ODI between the two groups. Symptoms in both groups improved significantly 2 weeks after surgery and during the final follow-up (*p* < 0.001 for all postoperative values compared with preoperative values). A statistically significant difference was found in the VAS of leg pain at 2 weeks postoperatively between the two groups. However, after examining the data distribution, the mean values of the two groups were similar. The observed difference may be due to the greater variability in the VAS of leg pain in the DLS group. Patients in the DLS group had a significantly higher VAS of back pain than those in the LSS group at the final follow-up (*p* = 0.039). Clinical satisfaction was achieved in 79 (81.4%) and 86 (89.7%) patients in the DLS and LSS groups, respectively, with no significant difference.

**Table 3:** Comparison of clinical outcomes between patients in the DLS and LSS groups.

	DLS group (*n* = 97)	LSS group (*n* = 97)	*p*
VAS for back pain			
Preoperative	40 (34–45)	34 (27–46)	0.084
Two weeks postoperative	15 (11–17)	14 (11–18)	0.422
Final follow-up	19 (12–29)	16 (12–20)	0.039*
VAS for leg pain			
Preoperative	57 (54–65)	59 (48–58)	0.723
Two weeks postoperative	13 (8–18)	16 (12–18)	0.024*
Final follow-up	12 (5–25)	18 (8–28)	0.143
ODI			
Preoperative	52 (48–58)	56 (44–64)	0.517
Two weeks postoperative	10 (8–15)	12 (9–16)	0.309
Final follow-up	16 (6–34)	15 (13–22)	0.110
Clinical satisfactory, *n* (%)	79 (81.4%)	86 (89.7%)	0.227

* Statistically significant at *p* < 0.05.

The main surgical complications were as follows: Among the 194 patients, two developed hematomas, one developed surgical site infection, 10 developed recurrent stenosis (nerve compression on the same side and at the same level as the initial onset of LSS), and 11 developed stenosis in other locations (contralateral side or other levels). There was no significant difference in the prevalence of surgical complications between the two groups (15.5% vs. 9.3%, *p* = 0.191, Table 4). In the DLS group, 11 of 97 patients (11.3%) and in the LSS group, 8 of 97 patients (8.2%) required reoperation, respectively.

**Table 4:** Summary of major surgical complications.

	DLS group	LSS group	*p*
Overall (*n*, %)	15 (15.5%)	9 (9.3%)	0.191
Recurrent stenosis (*n*)	4	6	
Stenosis in other locations (*n*)	9	2	
Hematoma formation (*n*)	1	1	
Surgical site infection (*n*)	1	0	

**Figure 3:**
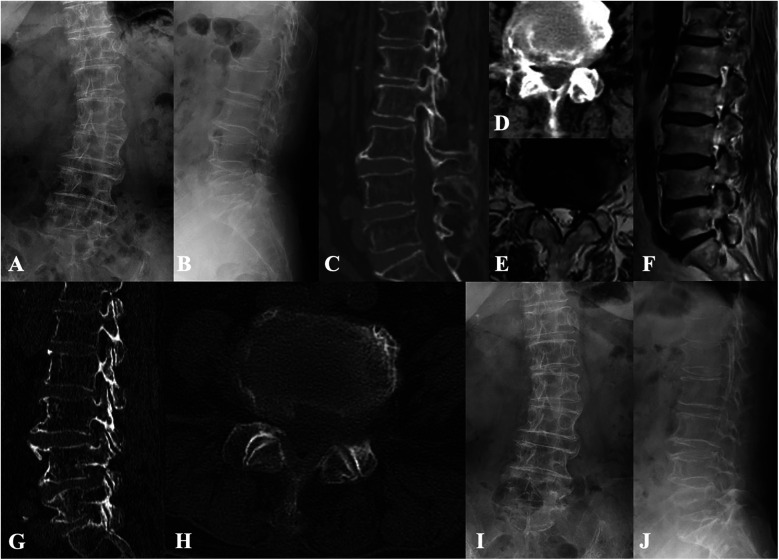
Case illustration of lumbar endoscopic decompression for the treatment of lumbar spinal stenosis and degenerative lumbar scoliosis. **(A,B)** Preoperative x-rays show degenerative lumbar scoliosis and vertebral osteophyte formation. **(C–F)** Preoperative CT and MR images show lateral recess and intervertebral foramen stenosis caused by compression from intervertebral discs and facet joints. **(G,H)** Postoperative CT images show adequate decompression while preserving the articular surfaces. **(I,J)** Radiological follow-up 3 years after surgery revealed no progression of lumbar scoliosis and no loss of intervertebral disc height.

The radiological findings before and after surgery are presented in Tables 5, 6, respectively. The mean radiological follow-up duration of patients in the DLS group was 27.1 ± 17.1 months, while that of patients in the LSS group was 28.7 ± 14.7 months (*p* = 0.632). Among these parameters, Cobb's angle decreased postoperatively (16.6 vs. 15.8, *p* = 0.045). Other parameters showed no significant difference between pre- and postoperative values in patients in the DLS group. Patients in the LSS group had a slight decrease in surgical level disc height postoperatively (8.3 vs. 8.1, *p* = 0.029). A case illustration of the pre- and postoperative imaging findings was provided in Figure 3.

**Table 5:** Comparison of pre- and postoperative imaging parameters of patients in the DLS group.

	Pre-operative	Post-operative	*p*
Cobb's angle (°)	16.6 ± 6.1	15.8 ± 6.3	0.045*
Segmental coronal angle (°)	5.1 ± 2.7	4.9 ± 2.9	0.652
Lumbar lordosis (°)	32.3 ± 12.7	32.2 ± 11.9	0.934
Surgical level disc height (mm)	7.5 ± 2.2	7.3 ± 2.2	0.112
Adjacent level disc height (mm)	7.4 ± 2.1	7.1 ± 2.1	0.077
L3 endplate angle (°)	5.7 ± 3.9	5.5 ± 4.1	0.596
L4 endplate angle (°)	6.8 ± 3.5	6.4 ± 3.5	0.386

* Statistically significant at *p* < 0.05.

**Table 6:** Comparison of pre- and postoperative imaging parameters of patients in the LSS group.

	Pre-operative	Post-operative	*p*
Cobb's angle (°)	2.9 ± 2.2	2.8 ± 1.9	0.721
Segmental coronal angle (°)	1.1 ± 1.4	1.0 ± 1.2	0.421
Lumbar lordosis (°)	37.7 ± 9.0	38.1 ± 8.2	0.512
Surgical level disc height (mm)	8.3 ± 1.8	8.1 ± 2.0	0.029*
Adjacent level disc height (mm)	8.2 ± 2.1	8.1 ± 2.2	0.059
L3 endplate angle (°)	1.2 ± 1.2	1.0 ± 1.2	0.094
L4 endplate angle (°)	1.0 ± 1.4	0.8 ± 1.0	0.393

* Statistically significant at *p* < 0.05.

## 4 Discussion

Patients with DLS are typically older and often have several comorbidities. For patients with mild deformities but severe radicular symptoms, it is critical to seek an appropriate approach for pain relief rather than correcting the deformity. In this retrospective study, the clinical and radiological outcomes of LSS patients with and without DLS were evaluated. Both groups benefited from a percutaneous transforaminal endoscopic procedure. Furthermore, after a mean radiological follow-up of more than 2 years, no significant deterioration was observed in the major radiological parameters. However, patients in the DLS group reported more severe low back pain than those in the LSS group did during the final follow-up. Compared to previous studies, this study had a relatively larger sample size and a longer follow-up duration, and it concluded that lumbar endoscopy can be a safe and effective treatment for LSS combined with mild DLS.

In recent years, numerous studies have shown that fusion and internal fixation may not be necessary in the treatment of various types of LSS. In contrast, decompression alone, particularly under endoscopic techniques, offers advantages such as enhanced safety, faster recovery, and cost-effectiveness (8, 15–17). However, there is still debate regarding the optimal surgical interventions of LSS with DLS. In a recent study, Khalifé (7) compared several surgical techniques and concluded that long fusion can result in better and more sustained clinical improvement. However, some experts believe that lumbar fixation may face difficulties in fully correcting sagittal imbalance and reconstructing lumbar lordosis in patients with DLS. Therefore, the improvement in patient symptoms may be limited (18). In another cadaver study, Rustenburg (19) also questioned the use of posterior instrumentation for treating DLS because it resulted in a rigid construct that could worsen lumbar degeneration. Recently, some researchers have embraced the concept of minimally invasive procedures. They reported that endoscopic or microscopic lumbar decompression surgeries can also provide satisfactory results for treating LSS with DLS (10, 20, 21), which is consistent with our results. Lumbar endoscopy has the advantage of providing detailed and adequate decompression of nerve roots, making it appropriate for foraminal and lateral recess stenosis associated with DLS.

However, DLS complicates lumbar endoscopic procedures compared with treating LSS alone. Before surgery, preoperative imaging should be used to rule out significant lumbar instability. Then, it is critical to determine the responsible level. The L4 and L5 nerve roots are the most commonly affected, but collapse of the intervertebral foramen on the concave side, as well as vertebral rotation and displacement, are also common causes of nerve root compression at other levels (3). Thus, preoperative diagnostic nerve root blocks are typically required. Intraoperatively, the primary challenge is the collapse of the intervertebral foramen due to DLS. In patients with concave side foraminal stenosis, the foramen can be opened by positioning the body in a moderately contralateral bending position. During nerve root decompression, bony foraminoplasty is frequently performed to provide adequate space. Current evidence suggests that superior facet joint removal should be performed from ventral to dorsal, with a resection range of no more than 50% and no exposure of the articular surface (22). However, surgeons should avoid pursuing excessive facet joint protection, at the expense of reducing the effectiveness of nerve root decompression. When there is insufficient space on the dorsal side of the foramen, ventral decompression can be used, which involves removing osteophytes from the vertebral posterior margin. However, reducing vertebral notches is not recommended. This is due to the difficulty in removing the hard cortical bone, which can have a significant impact on local stress (23).

Furthermore, during the reduction of soft tissues, geriatric patients with LSS and DLS often exhibit the following characteristics. First, due to prolonged compression, the herniated discs are often fragmented or calcified. Careful removal under endoscopic procedures is necessary to avoid residual fragments that could fail to alleviate nerve root symptoms. Second, the anatomical structures in these patients are typically less distinct. The nerve roots are often adhered to the ligamentum flavum or scar tissue. Forceful separation must be avoided to prevent nerve root or dural sac injury. Proper elevation of the pressure of lavage fluid, use of adrenaline, and timely application of radiofrequency hemostasis can help provide a clear field of view and reduce blind operation during decompression. Third, the morphology of the Kambin's triangle may change as a result of local deformity, and the distance between the exiting and traversing nerve roots may be extremely close. Careful differentiation and protection of the exiting nerve root are critical. During the surgery under local anesthesia, the patient's real-time feedback must be carefully monitored to reduce the risk of nerve damage. Therefore, such surgeries require the expertise of experienced spinal surgeons.

Based on the above experience, the follow-up results of this study show that the majority of patients with LSS and DLS who underwent lumbar endoscopic decompression can achieve significant relief, with an efficacy comparable to that of patients without DLS. Furthermore, the reported rates of complications and reoperations in this study were both lower than previously documented rates [14.0%–38.7% for complications (10, 24–26) and 12.3%–41.0% for reoperations (27–29)] associated with decompression and fusion surgery for DLS. This conclusion is supported by similar findings from previous studies (20, 21, 30), indicating that lumbar endoscopic surgery can be a safe and effective treatment for LSS with DLS.

This study also reported the radiological outcomes of lumbar endoscopy for the treatment of LSS with DLS. The results showed that after an average of 27 months postoperatively, Cobb's angle improved slightly compared to preoperative values, whereas other parameters, including disc height of the surgical and adjacent level, lumbar lordosis, and L3 and L4 endplate angles, did not show statistically significant differences (Figure 1). This conclusion is supported by previous research. First, biomechanical studies have shown that limited foraminoplasty does not impair lumbar stability, range of motion, or stress (31). Second, unlike traditional open surgery, the surgical approach of lumbar endoscopy does not involve the detachment of posterior supporting structures such as the paravertebral muscles, allowing the lumbar spine to stabilize to a large degree. Third, while lumbar endoscopy cannot directly correct the deformity, postoperative symptom relief can help improve patients' postural abnormalities and thus their lumbar balance (13). Previous studies have examined the radiographic outcomes of decompression and fusion in treating LSS with DLS (7, 10). Despite its benefits in correcting deformities, it is estimated that 9%–41% of patients may develop adjacent segment degeneration postoperatively (32, 33). Additionally, open decompression can lead to iatrogenic spondylolisthesis, caused by defects in posterior spinal structures. Our radiographic follow-up indicated that lumbar endoscopic decompression may help to avoid the aforementioned issues.

However, lumbar endoscopy has some limitations. At the final follow-up, patients in the DLS group reported more severe low back pain than those in the LSS group did. Similar concerns were expressed by Ogura (34) and Chang (35), although the exact cause of this phenomenon is unknown. We propose that, despite compensatory changes such as facet hypertrophy that address instability in DLS patients, their lumbar spine cannot be considered “stable” when compared to those without DLS. Weakness in the paraspinal muscles and vertebral displacement in the sagittal or coronal planes significantly increase the risk of low back pain in DLS patients (3). On the other hand, factors such as osteoporosis, facet joint arthritis, excessive load on intervertebral discs, and the release of inflammatory factors can all contribute to low back pain (4, 30). Furthermore, while the results showed no statistical difference, more patients in the DLS group experienced postoperative degeneration at other levels, necessitating additional surgical intervention. We believe that this phenomenon is not the same as adjacent segment disease after lumbar instrumentation, but rather an acceleration of natural degeneration caused by preexisting multilevel intervertebral disc degeneration and uneven loading in DLS patients. Therefore, the current evidence does not support the suitability of endoscopic decompression for all DLS patients. For those with severe deformity or higher functional demands, fixation and fusion may still be necessary.

The authors consider that the following factors contribute to unsatisfactory postoperative outcomes in LSS and DLS patients undergoing lumbar endoscopic decompression: First, severe scoliosis may impair surgical efficacy. Previous guidelines stated that a Cobb angle greater than 30° should be considered a contraindication for pure decompression surgery (2, 36). In this study, there were six cases of patients with a Cobb angle >25° who, due to poor general conditions or concerns about surgical risks, were unable to undergo internal fixation surgery. Only one of these patients reported an excellent surgical outcome, while the others required additional treatments due to repeated low back or leg pain. In these patients, severe deformities cause deformation of the intervertebral spaces and excessive load on supporting structures, such as the paraspinal muscles, which are beyond the scope of spinal endoscopy treatment. Furthermore, these patients have a faster rate of lumbar degeneration than those without significant deformities, which cause symptoms on the contralateral side or in other segments. Second, patients with concurrent spondylolisthesis, vertebral rotation, or endplate degeneration may experience poor postoperative outcomes. Although these factors alone may not be absolute contraindications for endoscopic decompression, the presence of multiple deformities may indicate increased local inflammatory reactions and potential spinal instability. Third, intraoperative exploration that reveals severe disc degeneration with calcification, significant nerve root adhesions, or pannus formation may indicate a higher risk for poor postoperative outcomes. However, these theories are primarily based on the clinical experience of the operators, and the authors intend to clarify the risk factors influencing the surgical outcomes of patients with LSS and DLS in future studies.

This study has several limitations. First, it is a single-center retrospective study with a relatively small sample size, which may have introduced a selection bias. Second, the control group was established based on surgery date matching. Patients in the DLS group had a higher prevalence of cardiovascular disease and more frequent stenosis at L3/4 and L4/5 due to the presence of DLS. This reflects the characteristics of the study population, where patients with poor general health and higher anesthesia risks tended to undergo endoscopic decompression. Although previous studies suggested these differences may not directly affect surgical outcomes (37, 38), a more rigorously designed prospective cohort study is required in the future. Third, this study did not directly compare various surgical procedures, such as decompression combined with fusion or other minimally invasive decompression procedures, for treating LSS with DLS. Thus, the optimal treatment strategy for such patients requires further research.

## 5 Conclusion

Lumbar endoscopic decompression under local anesthesia for treating LSS with DLS can achieve generally satisfactory outcomes without causing further progression of deformity postoperatively. However, persistent postoperative back pain remains a significant challenge for this technique. Therefore, it can be considered an alternative to traditional lumbar fusion surgery, particularly for DLS patients with compromised overall health. Further research is needed to to determine the appropriate surgical indications.

## Data Availability

The raw data supporting the conclusions of this article will be made available by the authors, without undue reservation.
